# ﻿Morphological characteristics and phylogenetic analyses revealed five new species (Basidiomycota) from Southwestern China

**DOI:** 10.3897/mycokeys.114.145368

**Published:** 2025-02-28

**Authors:** Ali Yang, Lu Wang, Yongjun Hu, Yingtao Jiang, Guiying Shi, Changlin Zhao

**Affiliations:** 1 College of Horticulture, Gansu Agricultural University, Lanzhou 730000, China Gansu Agricultural University Lanzhou China; 2 Department of Edible Fungi, Institute of Biology, Gansu Academy of Sciences, Lanzhou 730000, China Institute of Biology, Gansu Academy of Sciences Lanzhou China; 3 College of Forestry, Southwest Forestry University, Kunming 650224, China Southwest Forestry University Kunming China

**Keywords:** Biodiversity, molecular systematics, new taxa, taxonomy, wood-inhabiting fungi, Yunnan Province

## Abstract

In the present study, five new wood-inhabiting fungal species, *Conferticiumalbocremeum* (Stereaceae, Russulales), *Dendrocorticiopsisluteoalba* (Punctulariaceae, Corticiales), *Eichleriellabiluoxueshanensis* (Auriculariaceae, Auriculariales), *Gloeohypochniciumyunnanense* (Incertae sedis, Russulales), and *Punctularianigrodontea* (Punctulariaceae, Corticiales), collected from southern China, are proposed based on a combination of morphological features and phylogenetic evidence. *Conferticiumalbocremeum* is characterized by membranaceous and smooth basidiomata with white to cream surface and ellipsoid and verrucose basidiospores (9–11 × 5–7 µm); *Dendrocorticiopsisluteoalba* is characterized white to buff, membranaceous basidiomata and ellipsoid, thin-walled and smooth basidiospores (7–8 × 4.5–5.5 µm); *Eichleriellabiluoxueshanensis* is characterized by coriaceous and grandinioid basidiomata with buff to cinnamon-buff hymenophore and allantoid, thin-walled basidiospores (13.5–17.5 × 7–9 µm); *Gloeohypochniciumyunnanense* is characterized by buff to cream, coriaceous and smooth basidiomata and globose, thick-walled and warted basidiospores (10–12 × 10–11.5 µm), and *Punctularianigrodontea* is characterized by resupinate to effused-reflexed basidiomata and ellipsoid, thin-walled and smooth basidiospores (8.5–10 × 5–6 µm). Sequences of the internal transcribed spacers (ITS) and the large subunit (nLSU) of the nuclear ribosomal DNA (rDNA) markers of the studied samples were generated. Phylogenetic analyses performed based on the ITS+nLSUrDNA gene regions with the maximum likelihood, maximum parsimony, and Bayesian inference methods revealed that five new species belong to the genera *Conferticium*, *Dendrocorticiopsis*, *Eichleriella*, *Gloeohypochnicium*, and *Punctularia*. Descriptions, illustrations, phylogenetic analysis results, and a comparison with closely related taxa of the five new species are provided.

## ﻿Introduction

The kingdom of fungi is one of the most diverse groups of living organisms on earth; its members occur across a broad range of ecosystems, including extreme environments, with an estimated number of species in the range of 2–3 million (Bhunjun et al. 2022, 2024; Wijayawardene et al. 2022, 2024; Dong et al. 2024a; Hyde et al. 2024a, b). Based on molecular phylogenetic evidence, numerous new taxa have been discovered in the past ten years (Wang and Cai 2023; Yuan et al. 2023; Zhao et al. 2023; Deng et al. 2024b; Dong et al. 2024b; He et al. 2024; Qin et al. 2024).

The genus *Conferticium* Hallenb. (Stereaceae, Russulales) was erected in 1980 and typified by *C.insidiosum* (Bourdot & Galzin) Hallenb., which is characterized by the resupinate basidiomes with membranaceous to ceraceous, smooth to tuberculate hymenophore, a monomitic simple-septate hyphae, and the numerous cylindrical, sinuous gloeocystidia (Bernicchia and Gorjón 2010). Based on the MycoBank database (http://www.mycobank.org, accessed on 27 January 2025) and the Index Fungorum (http://www.indexfungorum.org, accessed on 27 January 2025), six specific and infraspecific names were registered in *Conferticium*, and it is a small genus only including five widely recognized species up to now.

The genus *Dendrocorticiopsis* Sheng H. Wu et al. (Punctulariaceae, Corticiales) was introduced by Sheng H. Wu, with the description of one species, *D.orientalis* Sheng H. Wu et al. (Wei et al. 2022). It is conventionally defined by having strictly resupinate basidiomata, an ivory hymenophore, a monomitic hyphal system with clamped hyphae, encrusted cystidia, dendrohyphidia, and ellipsoid to ovoid basidiospores (Wei et al. 2022). Based on the MycoBank database (http://www.mycobank.org, accessed on 27 January 2025) and the Index Fungorum (http://www.indexfungorum.org, accessed on 27 January 2025), *Dendrocorticiopsis* is a monotypic genus.

The genus *Eichleriella* Bres. was introduced in 1903, typified by *E.incarnata* Bres., and it is a species-rich genus that belongs to Auriculariaceae (Auriculariales). The genus is characterized by annual or short-living perennial, leathery to ceraceous basidiomata with smooth, pale-colored hymenophore (in some species covered by spines), a monomitic to dimitic hyphal system with clamped genitive hyphae, cystidia often present, longitudinally septate basidia with 2- or 4-celled, and colorless, cylindrical to narrowly cylindrical basidiospores (Malysheva and Spirin 2017; Li et al. 2023; Deng et al. 2024a). Based on the MycoBank database (http://www.mycobank.org, accessed on 27 January 2025) and the Index Fungorum (http://www.indexfungorum.org, accessed on 27 January 2025), the genus *Eichleriella* has 32 specific and registered names, with 22 species accepted worldwide (Malysheva and Spirin 2017, Liu et al. 2019, Li et al. 2023; Deng et al. 2024a).

The genus *Gloeohypochnicium* (Parmasto) Hjortstam (Russulales), typified by *G.analogum* (Bourdot & Galzin) Hjortstam (Bernicchia and Gorjón 2010), is characterized by the resupinate basidiomes with smooth to tuberculate hymenophore, a monomitic hyphal system with clamps on generative hyphae, the numerous cylindrical, sinuous gloeocystidia, and globose to ellipsoid, thick-walled, warted basidiospores (Bernicchia and Gorjón 2010). Based on the MycoBank database (http://www.mycobank.org, accessed on 27 January 2025) and the Index Fungorum (http://www.indexfungorum.org, accessed on 27 January 2025), *Gloeohypochnicium* has registered two specific and infraspecific names, and it is a small genus only including two widely recognized species so far (Bernicchia and Gorjón 2010; He et al. 2024).

*Punctularia* Patouillard (Punctulariaceae, Corticiales) was typified with *P.tuberculosa* (Pat.) Pat. & Lagerh. (current name *P.atropurpurascens* (Berk. & Broome) Petch, which is characterized by resupinate to effused-reflexed basidiomata, gelatinous when fresh, rigid upon drying, tuberculate or radial ridges hymenophore, a monomitic hyphal system with clamped generative hyphae, yellowish to brown dendrohyphidia, and thin-walled, smooth, ellipsoid, acyanophilous basidiospores (Bernicchia and Gorjón 2010). Based on the MycoBank database (http://www.mycobank.org, accessed on 27 January 2025) and the Index Fungorum (http://www.indexfungorum.org, accessed on 27 January 2025), *Punctularia* has registered six specific and infraspecific names, and three species have been recognized worldwide up to now (Bernicchia and Gorjón 2010; Guan et al. 2021; He et al. 2024).

Molecular phylogenetic approaches have revolutionized the fungal taxonomy of Basidiomycota in the last decades, and these advances have greatly enhanced our knowledge of species diversity in Basidiomycota (Lücking et al. 2021; He et al. 2022; Wang et al. 2023; Dong et al. 2024a). The family Auriculariaceae is the largest and best-supported clade in the order Auriculariales and consists of a large group of wood-decaying fungi with varied basidiomes (Dong et al. 2024b; He et al. 2024). Corticioid and stereoid taxa are numerous in Auriculariaceae and are typically classified into three main genera: *Eichleriella* Bres., *Exidiopsis* (Bref.) A. Møller, and *Heterochaete* Pat. (Malysheva and Spirin 2017; Li et al. 2023; Deng et al. 2024a). DNA sequence-based classification and identification of the genus *Eichleriella* (Auriculariaceae) have reported that six species have been described from China recently (Li et al. 2023; Deng et al. 2024a). Corticiales K.H. Larss. is a small order of corticioid fungi with four families *viz.*Corticiaceae Herter, Dendrominiaceae Ghobad-Nejhad, Punctulariaceae Donk, and Vuilleminiaceae Maire ex Lotsy (Wei et al. 2022; He et al. 2024). Most of the previous studies of Punctulariaceae focused on European species (Bernicchia and Gorjón 2010; Gorjón and Bernicchia 2017). In China, the research on this family mainly focuses on the genera *Dendrocorticiopsis* and *Punctularia*; some new taxa have been proposed based on a combination of morphological features and molecular data (Guan et al. 2021; Wei et al. 2022; He et al. 2024). Members of the Russulales Kreisel ex P.M. Kirk, P.F. Cannon & J.C. David exhibit diverse basidiome structures, which can range from agaricoid to discoid, clavarioid, polyporoid, corticoid, and even gasteroid, and the multigene phylogenetic analyses clarify the evolutionary relationships of some small genera in the latest study (Yuan et al. 2021; He et al. 2024). According to recent research in molecular systematics, the genus *Conferticium* (Stereaceae, Russulales) has reported one new species, *C.fissuratum* Xin Yang & C.L. Zhao from Yunnan Province (Bernicchia and Gorjón 2010; Shen et al. 2024). The taxonomic status of the genus *Gloeohypochnicium* is unclear, and it was only placed in the order Russulales, and it is a mystery genus; no new taxa in the genus have been described for nearly a decade (He et al. 2022, 2024). However, the phylogeny of the genus *Gloeohypochnicium* is ambiguous due to a lack of molecular evidence and morphological data.

In this paper, we presented the morphological characteristics and multigene molecular analyses with ITS and nLSU DNA markers to support the taxonomy and phylogenetic position of five new species.

## ﻿Materials and methods

### ﻿Sample collection and herbarium specimen preparation

Fresh basidiomata growing on angiosperm branches were collected from Dehong, Diqing, Tengchong, and Xishuangbanna of Yunnan Province, P.R. China. The samples were photographed *in situ*, and fresh macroscopic details were recorded. Photographs were recorded using a Jianeng 80D camera (Tokyo, Japan). Specimens were dried in an electric food dehydrator at 45 °C (Hu et al. 2022), then the specimens were sealed in an envelope and zip-lock plastic bags and labeled (Zhao et al. 2023). The dried specimens were deposited in the herbarium of the Southwest Forestry University (SWFC), Kunming, Yunnan Province, China.

### ﻿Morphology

The macromorphological descriptions were based on field notes and photos captured in the field and lab. The color terminology follows Petersen (1996). The micromorphological data were obtained from the dried specimens observed under a Nikon Eclipse E100 light microscope following Zhao and Wu (2017). The following abbreviations are used: KOH = 5% potassium hydroxide water solution, CB– = acyanophilous, IKI+ = amyloid, IKI– = both inamyloid and indextrinoid, L = mean spore length (arithmetic average for all spores), W = mean spore width (arithmetic average for all spores), Q = variation in the L/W ratios between the specimens studied, and n = a/b (number of spores (a) measured from given number (b) of specimens).

### ﻿DNA extraction, PCR, and sequencing

The CTAB rapid plant genome extraction kit-DN14 (Aidlab Biotechnologies Co., Ltd., Beijing, China) was used to obtain genomic DNA from the dried specimens according to the manufacturer’s instructions. The nuclear ribosomal internal transcribed spacer (ITS) region was amplified with the primer pair ITS5/ITS4 (White et al. 1990). The nuclear large subunit (nLSU) region with the primer pair LR0R/LR7 (Vilgalys and Hester 1990). The PCR procedure for ITS was as follows: initial denaturation at 95 °C for 3 min, followed by 35 cycles at 94 °C for 40 s, 58 °C for 45 s, and 72 °C for 1 min, and a final extension of 72 °C for 10 min. The PCR procedure for nLSU was as follows: initial denaturation at 94 °C for 1 min, followed by 35 cycles at 94 °C for 30 s, 48 °C for 1 min, and 72 °C for 1.5 min, and a final extension of 72 °C for 10 min. The PCR products were purified and sequenced at Kunming Tsingke Biological Technology Limited Company (Yunnan Province, P.R. China). All newly generated sequences were deposited in GenBank (Table 1).

**Table 1.** Names, voucher numbers, references, and corresponding GenBank accession numbers of the taxa used in the phylogenetic analyses. [* indicates type materials; – indicates sequence unavailability].

Taxa	Voucher no.	Locality	GenBank accession no.		References
			ITS	nLSU	
*Acanthobasidiumbambusicola*	He2357	China	KU559343	KU574833	Tian et al. 2018
*Acanthobasidiumphragmitis*	CBS 233.86	France	–	AY039305	Wu et al. 2001
*Acanthophysiumbisporum*	T614	USA	–	AY039327	Maekawa et al. 2023
*Acanthophysiumlividocaeruleum*	FP-100292	USA	–	AY039319	Maekawa et al. 2023
*Adustochaetepunctata*	CLZhao 29675	China	PP852052	PP849035	Dong et al. 2024b
*Aleurobotrysbotryosus*	He2712	China	KX306877	KY450788	Tian et al. 2018
*Aleurodiscusbambusinus*	He4261	China	KY706207	KY706219	Tian et al. 2018
*Alloexidiopsisaustraliensis*	LWZ 20180514-18	China	OM801934	OM801919	Liu et al. 2022
*Alloexidiopsiscalcea*	LWZ 20180904-14	China	OM801935	OM801920	Liu et al. 2022
*Alloexidiopsisgrandinea*	CLZhao 33798	China	PP852058	–	Dong et al. 2024b
*Alloexidiopsisxantha*	CLZhao 25093	China	PP852060	PP849040	Dong et al. 2024b
*Alloexidiopsisyunnanensis*	CLZhao 8106	China	MT215569	MT215565	Guan et al. 2020
*Amphistereumleveilleanum*	FP-106715	USA	KX262119	KX262168	Malysheva and Spirin 2017
*Amphistereumschrenkii*	HHB 8476	USA	KX262130	KX262178	Malysheva and Spirin 2017
*Amylostereumchailletii*	NH 8031	–	AF506406	AF506406	Larsson and Larsson 2003
*Amylostereumlaevigatum*	NH 12863	–	AF506407	AF506407	Larsson and Larsson 2003
*Aporpiumcaryae*	Miettinen 14774	Finland	JX044145	–	Miettinen et al. 2012
*Aporpiumcaryae*	WD 2207	Japan	AB871751	AB871730	Sotome et al. 2014
*Artomycesniveus*	CLZhao 19094	China	OR094479	OR461459	Dong et al. 2024a
*Artomycesyunnanensis*	CLZhao 7118	China	OR094476	OR461461	Dong et al. 2024a
*Auriculariaauricula-judae*	JT 04	UK	KT152099	KT152115	Tohtirjap et al. 2023
*Auriculariacornea*	Dai 13621	China	MZ618936	MZ669905	Tohtirjap et al. 2023
*Auriculariamesenterica*	FO 25132	Germany	AF291271	AF291292	Weiß and Oberwinkler 2001
*Auriculariapolytricha*	TUFC 12920	Japan	AB871752	AB871733	Sotome et al. 2014
*Auriculariatibetica*	Dai 13336	China	MZ618943	MZ669915	Tohtirjap et al. 2023
*Australovuilleminiacoccinea*	BCP5551	New Zealand	HM046875	HM046930	Ghobad-Nejhad et al. 2010
*Basidiodeserticahydei*	SQUCC 15289	Oman	MW077150	MW077159	Wei et al. 2022
*Bondarzewiaoccidentalis*	AFTOL-ID 452	Canada	DQ200923	DQ234539	Zhou et al. 2021
*Bondarzewiapodocarpi*	Dai 9261	China	KJ583207	KJ583221	Zhou et al. 2021
*Conferticiumalbocremeum*	CLZhao 35693*	China	PQ197729	PQ783842	Present study
*Conferticiumalbocremeum*	CLZhao 36343	China	PQ783832	PQ783843	Present study
*Conferticiumalbocremeum*	CLZhao 37176	China	PQ783833	PQ783844	Present study
*Conferticiumalbocremeum*	CLZhao 39238	China	PQ783834	–	Present study
*Conferticiumfissuratum*	CLZhao 34654	China	PQ201856	–	Shen et al. 2024
*Conferticiumfissuratum*	CLZhao 34662	China	PQ201857	–	Shen et al. 2024
*Conferticiumheimii*	CBS321.66	African	AF506381	AF506381	Tian et al. 2018
*Conferticiumochraceum*	CLZhao 21515	China	ON211619	–	Present study
*Conferticiumochraceum*	G07_P24A	Switzerland	KT943933	–	Stroheker et al. 2018
*Conferticiumravum*	CBS:125849	Estonia	MH863805	MH875269	Vu et al. 2019
*Conferticiumravum*	NH13291	USA	AF506382	AF506382	Larsson and Larsson 2003
*Corticiumroseum*	MG252	China	MW805872	MW805836	Ghobad-Nejhad et al. 2021
*Corticiumthailandicum*	MG242	Tailand	MW805868	MW805831	Ghobad-Nejhad et al. 2021
*Cytidiasalicina*	MG49	Finland	GU590881	HM046921	Wei et al. 2022
*Dendrocorticiopsisluteoalba*	CLZhao 30380*	China	PQ783828	PQ783838	Present study
*Dendrocorticiopsisorientalis*	He 4195	China	MW580926	MW580921	Wei et al. 2022
*Dendrocorticiopsisorientalis*	WEI 20-166	China	MW580922	MW580924	Wei et al. 2022
*Dendrocorticiumpolygonioides*	CBS:106.56	France	MH857525	MH869062	Vu et al. 2019
*Dendrocorticiumroseocarneum*	KUC20121109-32	Korea	KJ668559	KJ668413	Ghobad-Nejhad and Duhem 2014
*Dendrominiadryina*	MG159	France	JX892936	JX892937	Ghobad-Nejhad and Duhem 2014
*Dendrominiaericae*	MG162	France	JX892938	JX892939	Ghobad-Nejhad and Duhem 2014
*Dentipelliculaaustroafricana*	Dai 12580	Africa	KJ855274	KJ855275	Zhou et al. 2021
*Dentipelliculataiwaniana*	Dai 10867	China	JQ349115	JQ349101	Zhou et al. 2021
*Dentipellopsisdacrydicola*	Dai 12004	–	JQ349104	JQ349089	Zhou and Dai 2013
*Dentipellopsisdacrydicola*	Dai 12010	–	–	JQ349090	Zhou and Dai 2013
*Disporotrichumdimorphosporum*	CBS:419.70	Netherland	MH859776	MH871538	Vu et al. 2019
*Disporotrichumdimorphosporum*	CBS:433.85	USA	MH861895	MH873584	Vu et al. 2019
*Eichleriellaalliciens*	He4055b	Thailand	MH178244	–	Li et al. 2023
*Eichleriellaalliciens*	HHB 7194	USA	KX262120	KX262169	Malysheva and Spirin 2017
*Eichleriellaalpina*	He 20120916-1	China	MH178245	MH178268	Li et al. 2023
*Eichleriellabactriana*	TAAM 104431	Uzbekistan	KX262138	KX262186	Malysheva and Spirin 2017
*Eichleriellabambusicola*	Dai 6391	China	MH178246	–	Li et al. 2023
*Eichleriellabiluoxueshanensis*	CLZhao 34516*	China	PQ783827	PQ783837	Present study
*Eichleriellabiluoxueshanensis*	CLZhao 34793	China	PQ787846	–	Present study
*Eichleriellacrocata*	He2969	China	MH178248	MH178271	Li et al. 2023
*Eichleriellacrocata*	TAAM 101077	Russia	KX262100	KX262147	Malysheva and Spirin 2017
*Eichlerielladelicata*	CLZhao 25143	China	PQ757163	–	Present study
*Eichlerielladelicata*	He3469	China	MH178250	MH178273	Li et al. 2023
*Eichlerielladesertorum*	LR 49350	Namibia	KX262142	KX262190	Malysheva and Spirin 2017
*Eichlerielladiscolor*	He4584	China	MH178252	MH178275	Li et al. 2023
*Eichlerielladiscolor*	He4763	China	MH178253	MH178276	Li et al. 2023
*Eichleriellaflavida*	LR 49412	UK	KX262137	KX262185	Malysheva and Spirin 2017
*Eichleriellaleucophaea*	LE 303261	Russia	KX262111	KX262161	Malysheva and Spirin 2017
*Eichleriellamacrospora*	He2189	USA	MH178251	MH178274	Li et al. 2023
*Eichleriellaochracea*	SP467242	Brazil	MK391514	–	Alvarenga et al. 2019
*Eichleriellashearii*	LR 23258	–	KX262139	–	Malysheva and Spirin 2017
*Eichleriellashearii*	USJ 54609	Costa Rica	AF291284	AF291335	Weiß et al. 2001
*Eichleriellasicca*	OM 17349	USA	KX262143	KX262191	Malysheva and Spirin 2017
*Eichleriellasinensis*	CLZhao 29368	China	PQ757164	–	Present study
*Eichleriellasinensis*	CLZhao 31647	China	PQ757165	PQ757166	Present study
*Eichleriellasinensis*	He4196	China	MH178254	MH178277	Li et al. 2023
*Eichleriellatenuicula*	CLZhao 35797	China	PQ197731	–	Present study
*Eichleriellatenuicula*	He3483	China	MH178256	MH178279	Li et al. 2023
*Eichleriellaxinpingensis*	CLZhao 836	China	MK560879	MK560883	Liu et al. 2019
*Eichleriellaxinpingensis*	CLZhao 842	China	MK560880	MK560884	Liu et al. 2019
*Eichleriellayunnanensis*	CLZhao 31317	China	PP889850	PP897009	Deng et al. 2024a
*Eichleriellayunnanensis*	CLZhao 31350	China	PP889852	PP897010	Deng et al. 2024a
*Elmerinacladophora*	Miettinen 14314	Indonesia	MG757509	MG757509	Malysheva et al. 2018
*Elmerinasclerodontia*	Miettinen 16431	Malaysia	MG757512	MG757512	Malysheva et al. 2018
*Erythriciumhypnophilum*	MG169	France	MW805858	MW805823	Ghobad-Nejhad et al. 2021
*Erythriciumlaetum*	MG72	–	GU590875	GU590878	Ghobad-Nejhad et al. 2021
*Exidiacandida*	VS 3921	Russia	KY801867	KY801892	Spirin et al. 2018
*Exidiaglandulosa*	MW 355	Germany	AF291273	AF291319	Weiß et al. 2001
*Exidiapithya*	MW 313	Germany	AF291275	AF291321	Weiß et al. 2001
*Exidiareflexa*	Dai 20833	China	MN850386	MN850362	Ye et al. 2020
*Exidiasubglandulosa*	Wu 270	China	MN850381	MN850357	Ye et al. 2020
*Exidiopsiseffusa*	OM 19136	Finland	KX262145	KX262193	Malysheva and Spirin 2017
*Gloeocystidiellumbisporum*	KHL11135	Norway	AY048877	AY048877	Larsson and Larsson 2003
*Gloeocystidiellumclavuligerum*	FCUG2731	Russia	AF310083	AF310083	Larsson and Larsson 2003
*Gloeodontiaeriobotryae*	Dai 12080	–	JQ349116	JQ349103	Zhou and Dai 2013
*Gloeodontiapyramidata*	LR15502	–	AF506446	AF506446	Larsson and Larsson 2003
*Gloeohypochniciumanalogum*	NZFS:4549	New Zealand	MH409974	–	Hood et al. 2018
*Gloeohypochniciumanalogum*	PDD:91626	New Zealand	GQ411521	–	Fukami et al. 2010
*Gloeohypochniciumyunnanense*	CLZhao 30018	China	PQ783830	PQ783840	Present study
*Gloeohypochniciumyunnanense*	CLZhao 30049*	China	PQ783831	PQ783841	Present study
*Gloeophyllumabietinum*	H 22988	Switzerland	JX524619	KC782733	He et al. 2014
*Hericiumabietis*	NH 6990	Canada	AF506456	AF506456	Zhou et al. 2021
*Hericiumcoralloides*	NH 282	Sweden	AF506459	AF506459	Zhou et al. 2021
*Heterobasidionannosum*	Dai 20962	China	ON417163	ON417213	Liu et al. 2022
*Heteroradulumaustraliense*	LWZ 20180512-25	Australia	MZ325255	MZ310425	Li et al. 2022
*Heteroradulumdegluben*	LE 38182	Sweden	KX262112	KX262162	Malysheva and Spirin 2017
*Heteroradulumkmetii*	He 4915	China	MH178262	MH178286	Li et al. 2023
*Heteroradulumlabyrinthinum*	Yuan 1600	China	KM379139	KM379140	Yuan et al. 2018
*Heteroradulummussooriense*	Dai 17193	China	MH178265	MH178289	Li et al. 2023
*Hyphodermacremeoalbum*	CLZhao 17007	China	OM985716	OM985753	Dong et al. 2024a
*Lactariuscrocatus*	KVP08034	Thailand	HQ318243	HQ318151	Wu et al. 2020
*Lactariusdeceptivus*	AFTOL-ID 682	USA	AY854089	AY631899	Wu et al. 2020
*Laetisariafuciformis*	CBS:182.49	Netherlands	MH856485	MH868023	Vu et al. 2019
*Laetisariaroseipellis*	CBS:299.82	–	EU622846	EU622844	Vu et al. 2019
*Lawreymycespalicei*	Palice 2509	Ecuador	AY542864	AY542864	Lücking and Moncada 2017
*Lawreymycespalicei*	Palice 4369	Ecuador	AY542865	AY542865	Lücking and Moncada 2017
*Lentinellussublineolatus*	TENN 059307	Austria	NR119505	–	Dong et al. 2024a
*Lentinellusvulpinus*	7267	Sweden	AY513230	–	Kneal and Smith 2015
*Marchandiomycesaurantioroseus*	FCUG 1166	Sweden	KP864659	HM046929	Ghobad-Nejhad et al. 2021
*Marchandiomycescorallinus*	JL128-98	–	AY583327	AY583331	DePriest et al. 2005
*Megalocystidiumdiffissum*	V.Spirin4244	Sweden	MT477147	MT477147	Spirin et al. 2021
*Megalocystidiumleucoxanthum*	HK9808	Sweden	AF506420	AF506420	Spirin et al. 2021
*Mycobernardiaincrustans*	CBS:172.36	Canada	MH855759	MH867272	Vu et al. 2019
*Mycobernardiaincrustans*	Duhem 3613	France	MW805860	MW805825	Ghobad-Nejhad et al. 2021
*Neoaleurodiscusfujii*	He2921	China	KU559357	KU574845	Dai et al. 2017
*Neoaleurodiscusfujii*	Wu0807-41	Japan	–	FJ799924	Dai et al. 2017
*Peniophorahalimi*	CBS:864.84	France	MH861845	MH873533	Vu et al. 2019
*Peniophoraincarnata*	CBS:398.50	France	MH856680	MH868197	Vu et al. 2019
*Protodaedaleafoliacea*	Yuan 5691	China	JQ764666	JQ764644	Zhou and Dai 2013
*Protodaedaleahispida*	WD 548	Japan	AB871768	AB871749	Sotome et al. 2014
*Punctulariaatropurpurascens*	UC 2022981	USA	KP814559	–	Knijn & Ferretti 2018
*Punctulariaatropurpurascens*	WEI 17-662	China	MW570883	MW570888	Wei et al. 2022
*Punctulariabambusicola*	CLZhao 4133	China	MW559982	MW559984	Guan et al. 2021
*Punctulariabambusicola*	CLZhao 9098	China	MW559983	MW559985	Guan et al. 2021
*Punctularianigrodontea*	CLZhao 30592*	China	PQ783829	PQ783839	Present study
*Punctulariastrigosozonata*	AFTOL-ID 1248	–	DQ398958	AF518642	Wei et al. 2022
*Punctulariastrigosozonata*	CBS:34534	–	MH855559	MH867064	Vu et al. 2019
*Punctulariopsisefibulata*	Burdsall 8824	USA	KR494276	KR494277	Wei et al. 2022
*Punctulariopsisobducens*	MG70	Ethiopia	HM046918	HM046933	Ghobad-Nejhad et al. 2010
*Punctulariopsissubglobispora*	FCUG 2535	Argentina	HM046917	HM046932	Guan et al. 2021
*Russulablennia*	569/BB08.066	Switzerland	MH545687	KU237556	Wu et al. 2020
*Russulapseudociliata*	545/BB08.061	Switzerland	MH545688	KU237537	Wu et al. 2020
*Sclerotremagriseobrunneum*	TN 2722	Canada	KX262144	KX262192	Malysheva and Spirin 2017
*Sclerotremagriseobrunneum*	VS 7674	Russia	KX262140	KX262188	Malysheva and Spirin 2017
*Scytinostromaacystidiatum*	Dai 24608	China	OQ689127	OQ629351	Zhang et al. 2023
*Scytinostromabambusinum*	JXH 643	China	OR510627	PP660873	Ji et al. 2024
*Sistotremabrinkmannii*	236	Netherlands	JX535169	JX535170	Alvarenga and Gibertoni 2021
*Stereodiscuspseudotrivialis*	SPG6799	Argentina	OR506747	OR506751	Gorjón and Greslebin 2024
*Stereodiscuspseudotrivialis*	SPG6874	Argentina	OR506744	OR506746	Gorjón and Greslebin 2024
*Stereumhirsutum*	CBS:108532	Russia	MH862810	MH874407	Vu et al. 2019
*Stereumsanguinolentum*	CBS:529.50	Canada	MH856746	MH868268	Vu et al. 2019
*Terrestriporiaalba*	Dai 18546	Malaysia	MT068562	MT068558	Wu et al. 2020
*Terrestriporiaalba*	Dai 18547	Malaysia	MT068563	MT068559	Wu et al. 2020
*Tremellochaeteatlantica*	URM90199	Brazil	MG594381	MG594383	Alvarenga et al. 2019
*Tremellochaetecilliata*	SP467241	Brazil	MK391523	MK391529	Alvarenga et al. 2019
*Tremellochaetejaponica*	LE 303446	Russia	KX262110	KX262160	Malysheva and Spirin 2017
*Varariafissurata*	CLZhao 8171	China	OQ025219	OR539503	Deng et al. 2024b
*Varariatropica*	CBS:704.81	France	MH861447	MH873189	Vu et al. 2019
*Vuilleminiacomedens*	AFTOL-ID 1247	–	DQ398959	AF518666	Wei et al. 2022
*Vuilleminiacoryli*	MG136	Turkmenistan	JN387996	JN388005	Ghobad-Nejhad and Ginns 2012
*Vuilleminiacystidiata*	KUC20131022-26	Korea	KJ668433	KJ668285	Wei et al. 2022
*Vuilleminiaerastii*	MG97	Canada	JN387998	JN388007	Ghobad-Nejhad and Ginns 2012
*Vuilleminiamacrospora*	MG167	France	JX892940	JX892941	Ghobad-Nejhad and Duhem 2014
*Vuilleminianilsii*	MG171	France	JX892947	JX892948	Ghobad-Nejhad and Duhem 2014
*Vuilleminiapseudocystidiata*	MG69	France	HM046888	HM046928	Ghobad-Nejhad et al. 2010
*Waiteacircinata*	CBS:472.82	USA	MH861518	MH873265	Vu et al. 2019
*Waiteaguianensis*	GUY13-110	Guiana	MW449090	MW449101	Wei et al. 2022
*Xylobolusfrustulatus*	He2231	USA	KU881905	KU574825	Tian et al. 2018
*Xylobolussubpileatus*	FP-106735	USA	–	AY039309	Tian et al. 2018

### ﻿Molecular phylogeny

**Figure 1.**
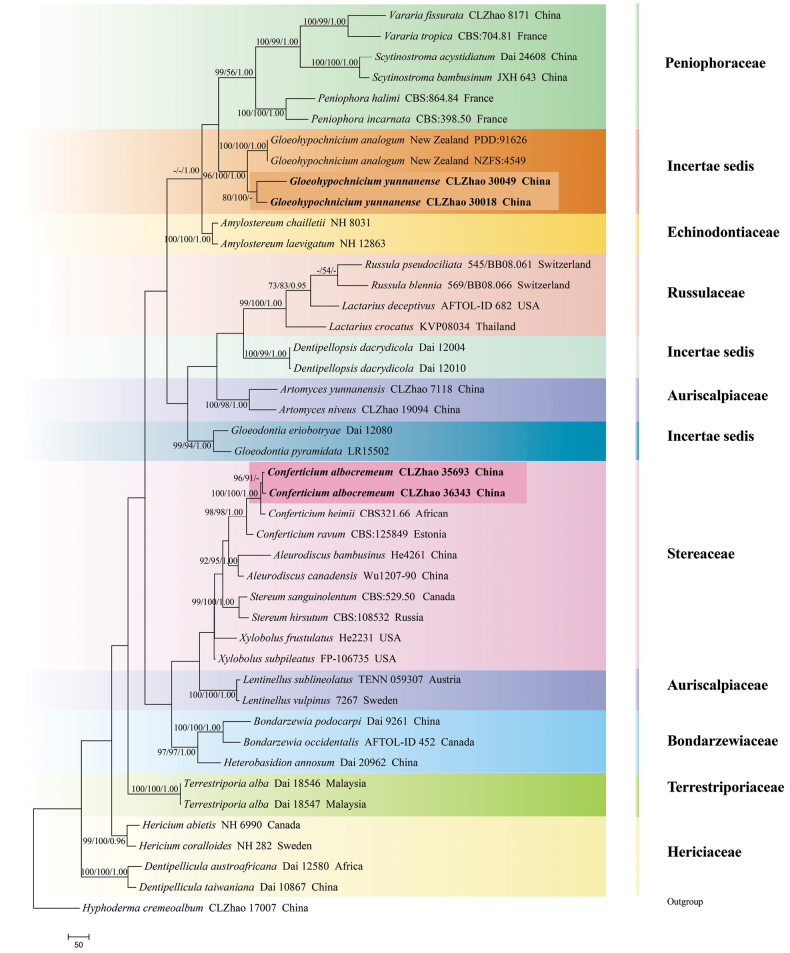
Maximum parsimony strict consensus tree illustrating the phylogeny of *Conferticium* and *Gloeohypochnicium* and related genera in the order Russulales, based on ITS+nLSU sequences; branches are labeled with maximum likelihood bootstrap value ≥ 70%, parsimony bootstrap value ≥ 50%, and Bayesian posterior probabilities ≥ 0.95.

**Figure 2.**
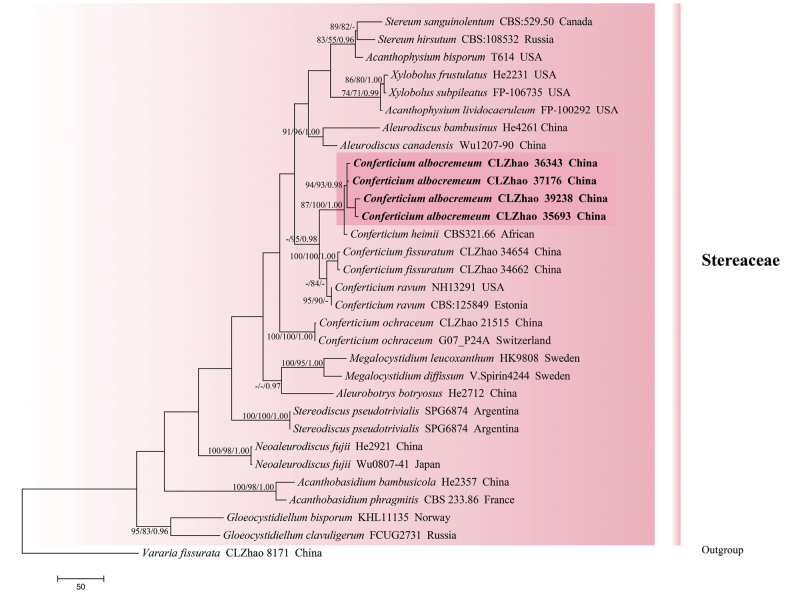
Maximum parsimony strict consensus tree illustrating the phylogeny of *Conferticium* and related genus in the family Stereaceae, based on ITS+nLSU sequences; branches are labeled with maximum likelihood bootstrap value ≥ 70%, parsimony bootstrap value ≥ 50%, and Bayesian posterior probabilities ≥ 0.95.

**Figure 3.**
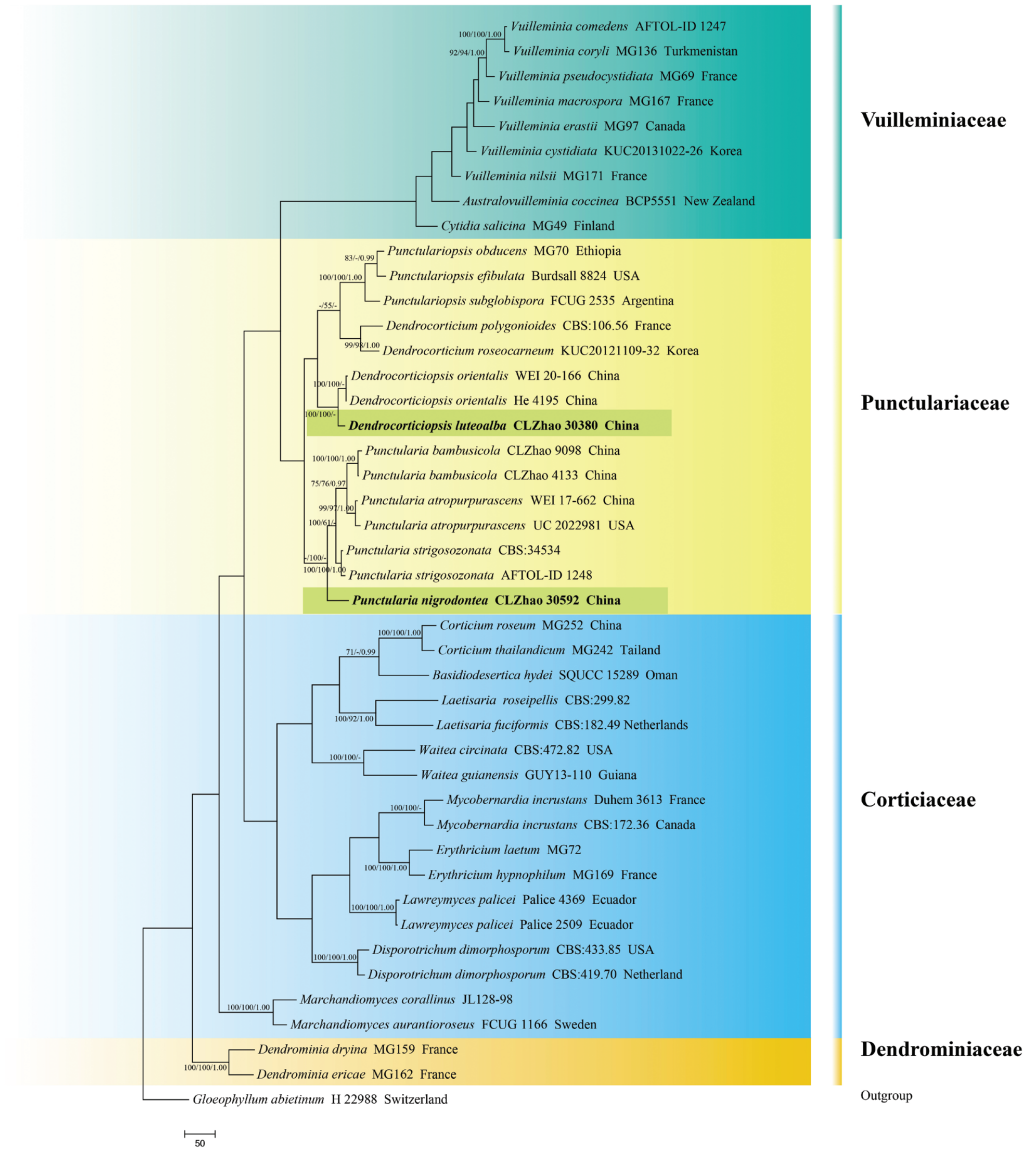
Maximum parsimony strict consensus tree illustrating the phylogeny of *Dendrocorticiopsis* and *Punctularia* and related genera in the order Corticiales, based on ITS+nLSU sequences; branches are labeled with maximum likelihood bootstrap value ≥ 70%, parsimony bootstrap value ≥ 50%, and Bayesian posterior probabilities ≥ 0.95.

**Figure 4.**
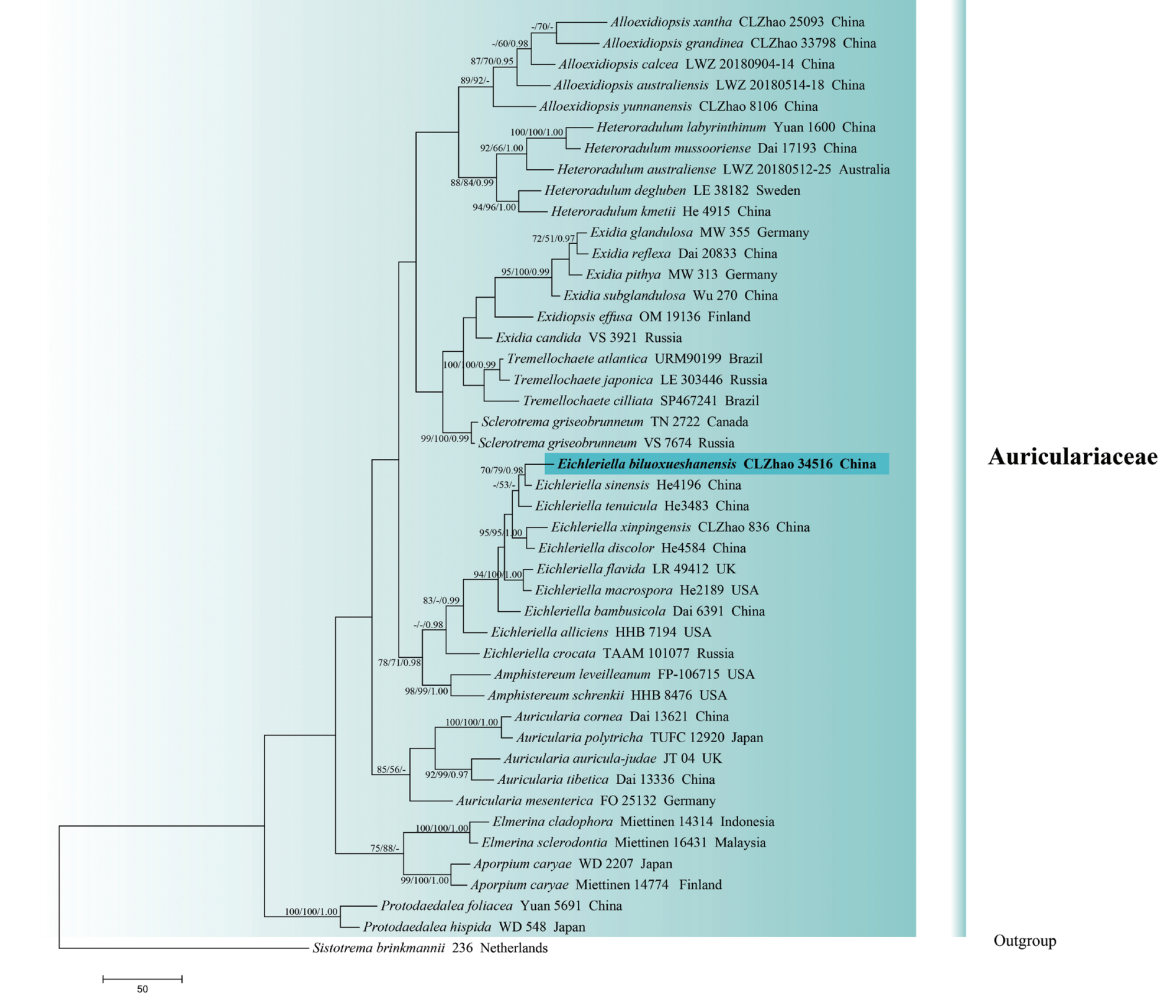
Maximum parsimony strict consensus tree illustrating the phylogeny of *Eichleriella* and related genera in the family Auriculariaceae, based on ITS+nLSU sequences; branches are labeled with maximum likelihood bootstrap value ≥ 70%, parsimony bootstrap value ≥ 50%, and Bayesian posterior probabilities ≥ 0.95.

**Figure 5.**
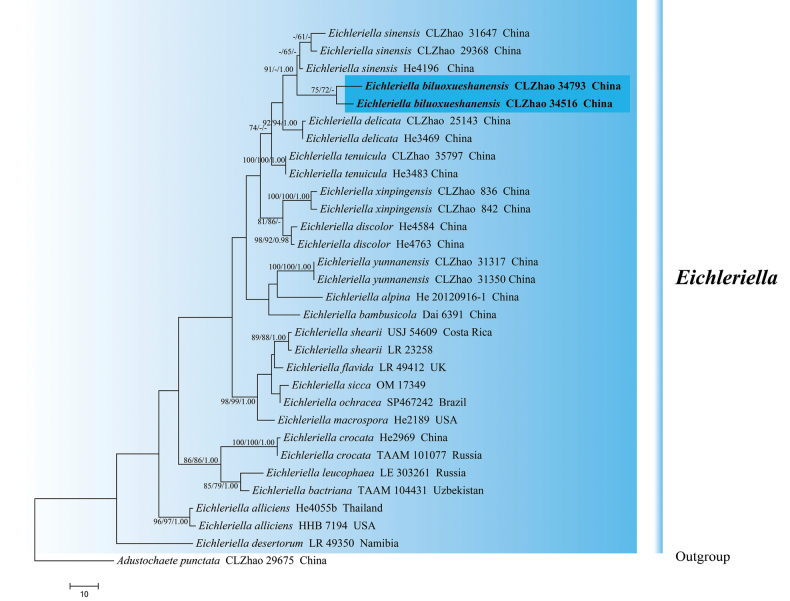
Maximum parsimony strict consensus tree illustrating the phylogeny of *Eichleriella* and related species in the genus *Eichleriella*, based on ITS+nLSU sequences; branches are labeled with maximum likelihood bootstrap value ≥ 70%, parsimony bootstrap value ≥ 50%, and Bayesian posterior probabilities ≥ 0.95.

The sequences were aligned in MAFFT version 7 using the G-INS-i strategy (Katoh et al. 2019). The alignment was adjusted manually using AliView version 1.27 (Larsson 2014). (1) *Hyphodermacremeoalbum* (Höhn. & Litsch.) Jülich was assigned as an outgroup to root trees in the ITS+nLSU analysis (Fig. 1) (Dong et al. 2024a); (2) *Varariafissurata* Y.L. Deng & C.L. Zhao were assigned as an outgroup to root trees following the ITS+nLSU analysis (Fig. 2) (Deng et al. 2024b); (3) *Gloeophyllumabietinum* (Bull.) P. Karst. was assigned as an outgroup to root trees following the ITS+nLSU analysis (Fig. 3) (He et al. 2014); (4) *Sistotremabrinkmannii* (Bres.) J. Erikss. was assigned as an outgroup to root trees following the ITS+nLSU analysis (Fig. 4) (Alvarenga and Gibertoni 2021); (5) *Adustochaetepunctata* J.H. Dong & C.L. Zhao were assigned as an outgroup to root trees following the ITS+nLSU analysis (Fig. 5) (Dong et al. 2024b).

Maximum parsimony (MP), maximum likelihood (ML), and Bayesian inference (BI) analyses were applied to the combined datasets following a previous study (Wu et al. 2022; Dong et al. 2024a), and the tree construction procedure was performed in PAUP* version 4.0b10 (Swofford 2002). All characters were equally weighted, and gaps were treated as missing data. Trees were inferred using the heuristic search option with TBR branch swapping and 1,000 random sequence additions. Max trees were set to 5000, branches of zero length were collapsed, and all parsimonious trees were saved. Clade robustness was assessed using bootstrap (BT) analysis with 1000 replicates (Felsenstein 1985). Descriptive tree statistics, tree length (TL), consistency index (CI), retention index (RI), rescaled consistency index (RC), and homoplasy index (HI) were calculated for each maximum parsimonious tree generated. The multiple sequence alignment was also analyzed using maximum likelihood (ML) in RAxML-HPC2 (Miller et al. 2012). Branch support (BS) for ML analysis was determined by 1000 bootstrap replicates.

MrModeltest 2.3 (Nylander 2004) was used to determine the best-fit evolution model for each dataset for Bayesian inference (BI), which was performed using MrBayes 3.2.7a with a general time reversible model of DNA substitution and a gamma distribution rate variation across sites (Ronquist et al. 2012). Four Markov chains were run twice from a random starting tree for 0.4 million generations of the datasets (Fig. 1), 0.4 million generations of the datasets (Fig. 2), 1.6 million generations of the datasets (Fig. 3), 0.8 million generations of the datasets (Fig. 4), and for 0.6 million generations of the datasets (Fig. 5), and the tree was sampled every 1000 generations. The first one-fourth of all generations were discarded as burn-in. The majority rule consensus tree of all remaining trees was calculated. Branches were considered as significantly supported if they received a maximum likelihood bootstrap value (BS) ≥70%, maximum parsimony bootstrap value (BT) ≥50%, or Bayesian posterior probabilities (BPP) ≥0.95.

## ﻿Results

### ﻿The phylogeny of *Conferticium*

The datasets based on ITS+nLSU (Fig. 1) comprise sequences from 44 fungal specimens representing 39 species. The datasets had an aligned length of 2,270 characters, of which 1,183 characters are constant, 293 are variable and parsimony-uninformative, and 794 are parsimony-informative. Maximum parsimony analysis yielded 1 equally parsimonious tree (TL = 4447, CI = 0.4160, H = 0.5840, RI = 0.5275, RC = 0.2194). Bayesian analysis and ML analysis resulted in a similar topology as MP analysis with an average standard deviation of split frequencies of 0.008678 (BI), and the effective sample size (ESS) across the two runs is double the average ESS (avg ESS) = 144.5. The phylogenetic tree (Fig. 1) inferred from ITS+nLSU sequences revealed that the new species *Conferticiumalbocremeum* grouped into the genus *Conferticium* and clustered into the family Stereaceae (Russulales), in which it was retrieved as a sister to *C.heimii* (Boidin) Sheng H. Wu.

The datasets based on ITS+nLSU (Fig. 2) comprise sequences from 31 fungal specimens representing 23 species. The datasets had an aligned length of 2,136 characters, of which 1,456 characters are constant, 298 are variable and parsimony-uninformative, and 382 are parsimony-informative. Maximum parsimony analysis yielded 1 equally parsimonious tree (TL = 1491, CI = 0.6237, HI = 0.3763, RI = 0.5681, RC = 0.3544). Bayesian analysis and ML analysis resulted in a similar topology as MP analysis with an average standard deviation of split frequencies of 0.013157 (BI), and the effective sample size (ESS) across the two runs is double the average ESS (avg ESS) = 265.5. The phylogenetic tree (Fig. 2) inferred from ITS+nLSU sequences revealed that *Conferticiumalbocremeum* grouped into the genus *Conferticium* and clustered into the family Stereaceae, in which it was closely related to *C.heimii*.

### ﻿The phylogeny of *Dendrocorticiopsis*

The datasets based on ITS+nLSU (Fig. 3) comprise sequences from 44 fungal specimens representing 37 species. The datasets had an aligned length of 2,181 characters, of which 1,313 characters are constant, 232 are variable and parsimony-uninformative, and 736 are parsimony-informative. Maximum parsimony analysis yielded 1 equally parsimonious tree (TL = 3423, CI = 0.4368, HI = 0.5632, RI = 0.5695, RC = 0.2488). Bayesian analysis and ML analysis resulted in a similar topology as MP analysis with an average standard deviation of split frequencies of 0.013154 (BI), and the effective sample size (ESS) across the two runs is double the average ESS (avg ESS) = 658. The phylogenetic tree (Fig. 1) inferred from ITS+nLSU sequences revealed that the new species *Dendrocorticiopsisluteoalba* grouped into the genus *Dendrocorticiopsis* and clustered into the family Punctulariaceae (Corticiales), in which it was retrieved as a sister to *D.orientalis* Sheng H. Wu et al.

### ﻿The phylogeny of *Eichleriella*

The datasets based on ITS+nLSU (Fig. 4) comprise sequences from 45 fungal specimens representing 43 species. The datasets had an aligned length of 2,104 characters, of which 1,516 characters are constant, 254 are variable and parsimony-uninformative, and 334 are parsimony-informative. Maximum parsimony analysis yielded 1 equally parsimonious tree (TL = 1783 CI = 0.4773, HI = 0.5227, RI = 0.5633, RC = 0.2688). Bayesian analysis and ML analysis resulted in a similar topology as MP analysis with an average standard deviation of split frequencies of 0.0024569 (BI), and the effective sample size (ESS) across the two runs is double the average ESS (avg ESS) = 361.5. The phylogenetic tree (Fig. 4) inferred from ITS+nLSU sequences revealed that *Eichleriellabiluoxueshanensis* grouped into the genus *Eichleriella* and clustered into the family Auriculariaceae, in which it was retrieved as a sister to *E.sinensis* (Teng) S.H. He & Nakasone.

The datasets based on ITS+nLSU (Fig. 5) comprise sequences from 31 fungal specimens representing 24 species. The datasets had an aligned length of 1,905 characters, of which 1,687 characters are constant, 101 are variable and parsimony-uninformative, and 117 are parsimony-informative. Maximum parsimony analysis yielded 1 equally parsimonious tree (TL = 402, CI = 0.6542, HI = 0.3458, RI = 0.7495, RC = 0.4094). Bayesian analysis and ML analysis resulted in a similar topology as MP analysis with an average standard deviation of split frequencies of 0.009368 (BI), and the effective sample size (ESS) across the two runs is double the average ESS (avg ESS) = 264.5. The phylogenetic tree (Fig. 5) inferred from ITS+nLSU sequences revealed that *Eichleriellabiluoxueshanensis* grouped into the genus *Eichleriella*, in which it was grouped with the clade comprising *E.sinensis* (Teng) S.H. He & Nakasone.

### ﻿The phylogeny of *Gloeohypochnicium*

The datasets based on ITS+nLSU (Fig. 1) comprise sequences from 44 fungal specimens representing 39 species. The datasets had an aligned length of 2,270 characters, of which 1,183 characters are constant, 293 are variable and parsimony-uninformative, and 794 are parsimony-informative. Maximum parsimony analysis yielded 1 equally parsimonious tree (TL = 4447, CI = 0.4160, HI = 0.5840, RI = 0.5275, RC = 0.2194). Bayesian analysis and ML analysis resulted in a similar topology as MP analysis with an average standard deviation of split frequencies of 0.008678 (BI), and the effective sample size (ESS) across the two runs is double the average ESS (avg ESS) = 144.5. The phylogenetic tree (Fig. 1) inferred from ITS+nLSU sequences revealed that *Gloeohypochniciumyunnanense* grouped into the genus *Gloeohypochnicium* and clustered into the order Russulales, in which it was closely related to *G.analogum* (Bourdot & Galzin) Hjortstam.

### ﻿The phylogeny of *Punctularia*

The datasets based on ITS+nLSU (Fig. 3) comprise sequences from 44 fungal specimens representing 37 species. The datasets had an aligned length of 2,181 characters, of which 1,313 characters are constant, 232 are variable and parsimony-uninformative, and 736 are parsimony-informative. Maximum parsimony analysis yielded 1 equally parsimonious tree (TL = 3423, CI = 0.4368, HI = 0.5632, RI = 0.5695, RC = 0.2488). Bayesian analysis and ML analysis resulted in a similar topology as MP analysis with an average standard deviation of split frequencies of 0.013154 (BI), and the effective sample size (ESS) across the two runs is double the average ESS (avg ESS) = 658. The phylogenetic tree (Fig. 1) inferred from ITS+nLSU sequences revealed that *Punctularianigrodontea* grouped into the genus *Punctularia* and clustered into the family Punctulariaceae (Corticiales), in which it was grouped with the clade comprising *P.atropurpurascens* (Berk. & Broome) Petch, *P.bambusicola* C.L. Zhao and *P.strigosozonata* (Schwein.) P.H.B. Talbot.

### ﻿Taxonomy

#### ﻿ Conferticium albocremeum

Taxon classificationFungiRussulalesStereaceae

L. Wang & C.L. Zhao sp. nov.

4A61C852-B8C1-514E-B3C8-39DA9CEBFA49

856958

Figs 6 , 7 , 8

##### Typification.

China. Yunnan Province • Xishuangbanna, Wild Elephant Valley, GPS coordinates: 22°10′N, 100°51′E, altitude: 900 m asl., on the fallen angiosperm branch, leg. C.L. Zhao, 25 January 2024 CLZhao 35693 (SWFC!).

##### Etymology.

*Albocremeum* (Lat.) refers to the new species having white to cream hymenophore.

##### Basidiomata.

Annual, resupinate, closely adnate, membranaceous, without odor or taste when fresh, up to 10 cm long, 2 cm wide, and 700 μm thick. Hymenophore smooth, white (60) to cream (4A2/3) when fresh, cream (4A2/3) upon drying. Sterile margin narrow, cream (4A2/3), up to 1 mm.

**Figure 6.**
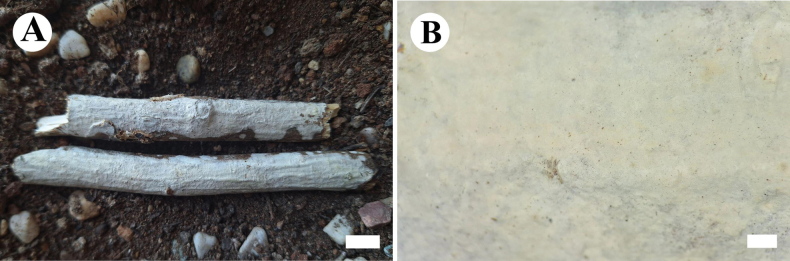
Basidiomata of *Conferticiumalbocremeum* (holotype CLZhao 35693). Scale bars: 1 cm (**A**); 1 mm (**B**).

##### Hyphal system.

Monomitic; generative hyphae with simple-septa, colorless, thin-walled, smooth, rarely branched, interwoven, IKI+, CB–, 2–3 µm in diameter; tissues unchanged in KOH.

**Figure 7.**
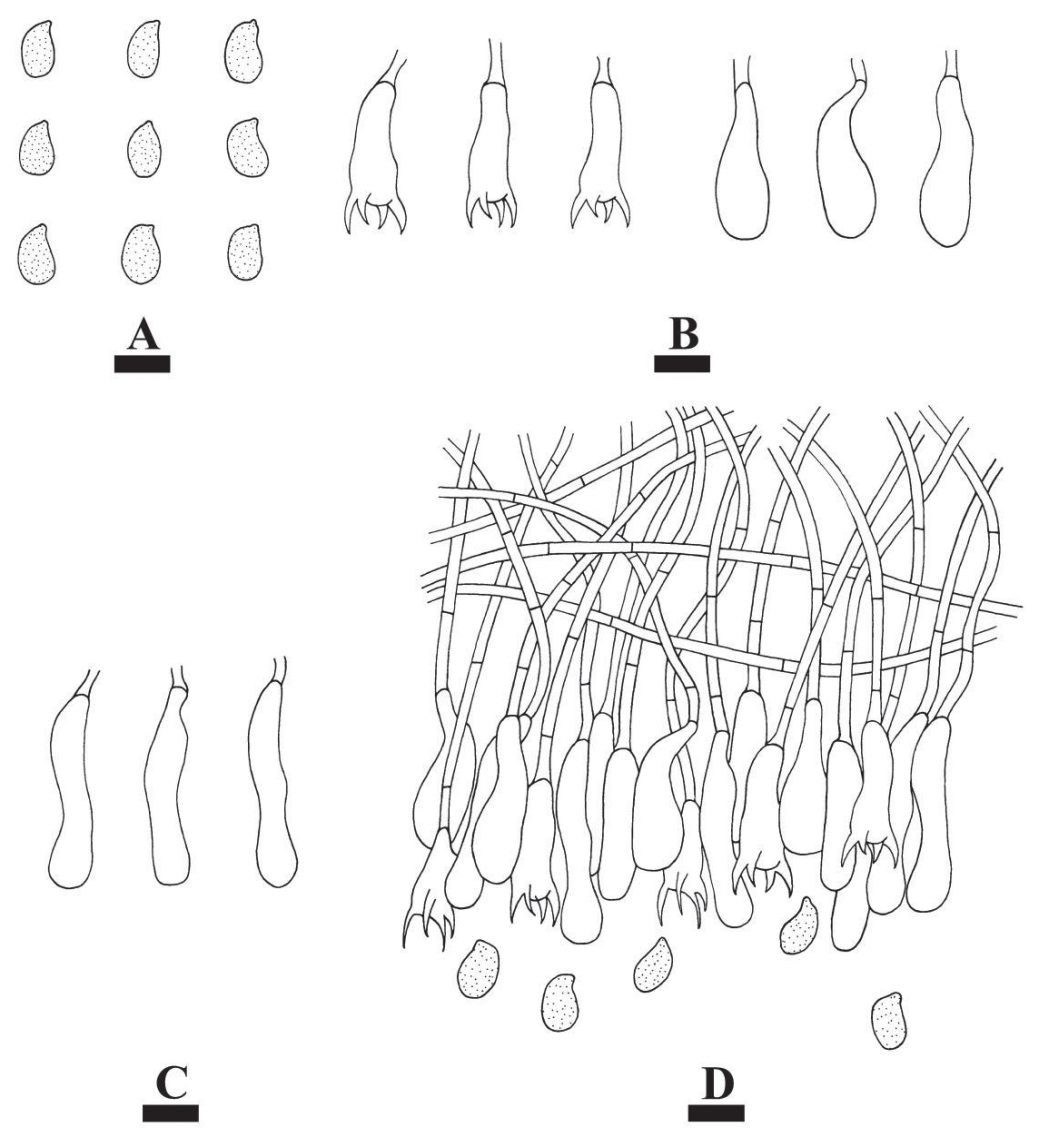
Microscopic structures of *Conferticiumalbocremeum* (holotype CLZhao 35693): basidiospores (**A**); basidia and basidioles (**B**); gloeocystidia (**C**); a section of the hymenium (**D**). Scale bars: 10 µm (**A–D**).

##### Hymenium.

Gloeocystidia subclavate, flexuous, colorless, mostly constricted in the middle, thin-walled, smooth, 33–47.5 × 5–8 µm. Basidia subcylindrical to subclavate, slightly flexuous, with a basal simple septum and four sterigmata, 22–36 × 4.5–7 µm; basidioles numerous, in shape similar to basidia.

**Figure 8.**
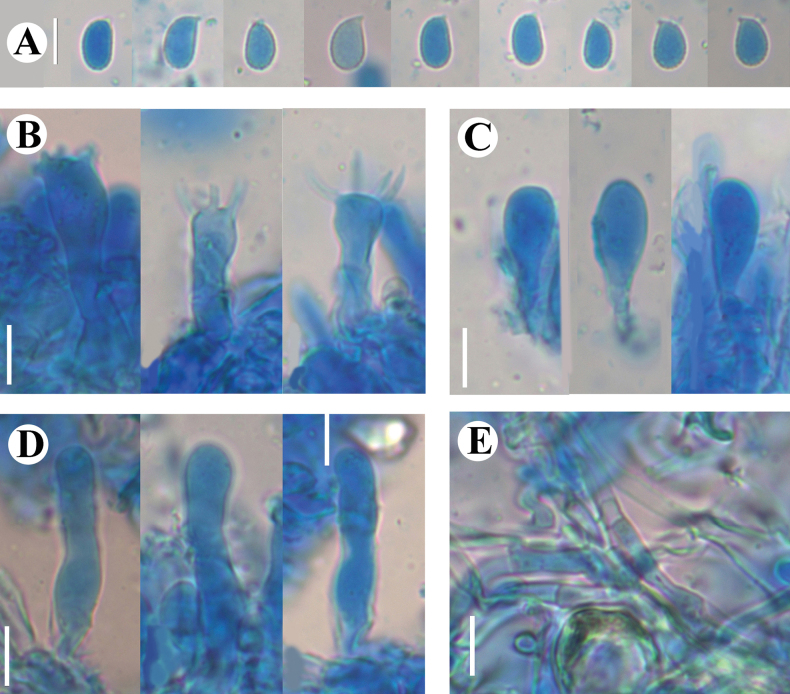
Sections of hymenium of *Conferticiumalbocremeum* (holotype CLZhao 35693): basidiospores (**A**); basidia (**B**); basidioles (**C**); gloeocystidia (**D**); a section of the generative hyphae (**E**). Scale bars: 10 µm (**A–E**); 10 × 100.

##### Spores.

Basidiospores ellipsoid with a distinct apiculus, colorless, thin-walled, finely verrucose but appearing smooth by light microscope, IKI+, CB–, 9–11 × 5–7 µm, L = 9.82 µm, W = 5.84 µm, Q = 1.36–1.68 (n = 90/3).

##### Additional specimens examined (paratypes).

China. Yunnan Province • Dehong, Mang City, Mengga Town, Tongbiguan Provincial Nature Reserve, GPS coordinates: 24°46′N, 97°34′E, altitude: 1300 m asl., on the fallen angiosperm branch, leg. C.L. Zhao, 29 June 2024, CLZhao 36343 • Dehong, Yingjiang County, Tongbiguan Provincial Nature Reserve, GPS coordinates: 25°50′N, 97°36′E, altitude: 1000 m asl., on the fallen angiosperm branch, leg. C.L. Zhao, 2 July 2024, CLZhao 37176 • Tengchong, Tuantian Town, Gaoligongshan National Nature Reserve, GPS coordinates: 25°27′N, 98°46′E, altitude: 2500 m asl., on the fallen angiosperm branch, leg. C.L. Zhao, 7 July 2024, CLZhao 39283 (SWFC!).

#### ﻿ Dendrocorticiopsis luteoalba

Taxon classificationFungiCorticialesPunctulariaceae

L. Wang & C.L. Zhao sp. nov.

3D3B5305-96D0-5FC0-AEDB-262A3FA24824

856959

Figs 9 , 10 , 11

##### Typification.

China. Yunnan Province • Dehong, Yingjiang County, Tongbiguan Provincial Nature Reserve, GPS coordinates: 25°50′N, 97°36′E, altitude: 1000 m asl., on the fallen angiosperm branch, leg. C.L. Zhao, 19 July 2023, CLZhao 30380 (SWFC!).

##### Etymology.

*Luteoalba* (Lat.) refers to the new species having a white to buff hymenophore.

##### Basidiomata.

Annual, resupinate, closely adnate, membranaceous, without odor or taste when fresh, up to 6 cm long, 2 cm wide, and 300 μm thick. Hymenophore smooth, white (60) when fresh, white (60) to buff (4A4) upon drying. Sterile margin narrow, white (60) to buff (4A4), up to 1 mm.

**Figure 9.**
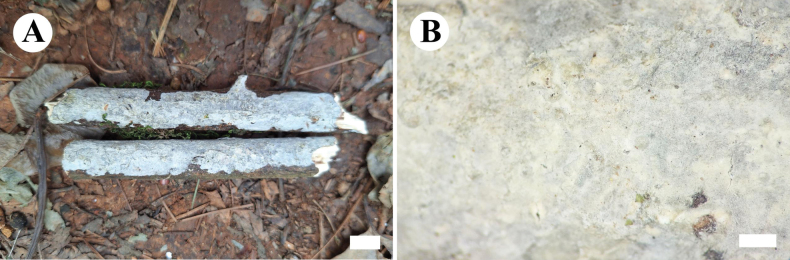
Basidiomata of *Dendrocorticiopsisluteoalba* (holotype CLZhao 30380). Scale bars: 1 cm (**A**); 1 mm (**B**).

##### Hyphal system.

Monomitic; generative hyphae with clamp connections, colorless, thin- to slightly thick-walled, smooth, branched, interwoven, usually with crystal masses, IKI–, CB–, 2.5–4 µm in diameter; tissues unchanged in KOH.

**Figure 10.**
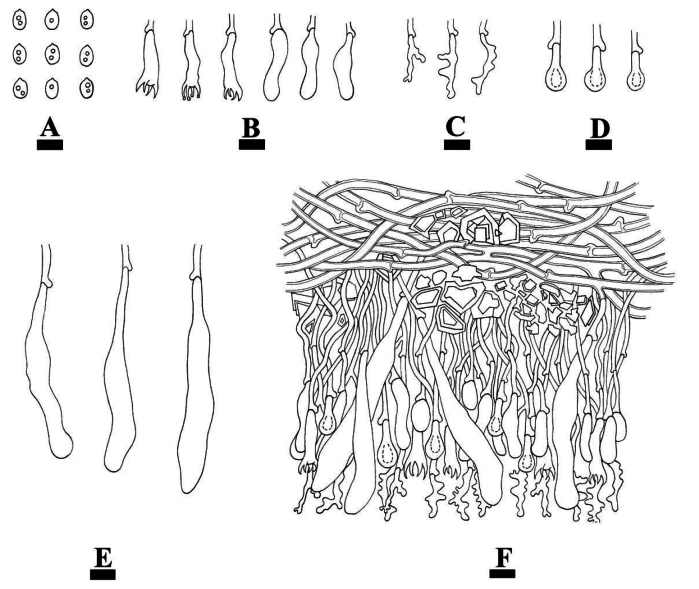
Microscopic structures of *Dendrocorticiopsisluteoalba* (holotype CLZhao 30380): basidiospores (**A**); basidia and basidioles (**B**); dendrohyphidia (**C**); cystidia (**D**); gloeocystidia (**E**); a section of the hymenium (**F**). Scale bars: 10 µm (**A–F**).

##### Hymenium.

Cystidia are of two types: (1) capitate, apically with resinous materials, gradually dissolving in KOH, colorless, thin-walled, smooth, 8.5–14 × 6.5–8.5 µm; (2) gloeocystidia, clavate to subulate, slightly flexuous, colorless, thin-walled, smooth, 68.5–90 × 8–10 µm. Dendrohyphidia numerous, thick-walled toward base, colorless, 16–19 × 2.5–3.5 µm. Basidia subclavate to clavate, flexuous, with a basal clamp connection and four sterigmata, 15.5–28.5 × 4–5.5 µm; basidioles numerous, in shape similar to basidia.

**Figure 11.**
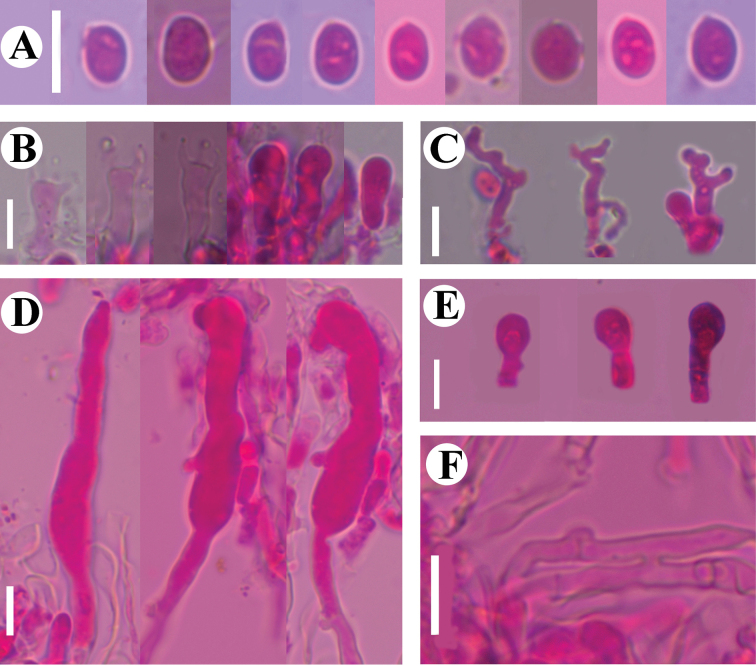
Sections of hymenium of *Dendrocorticiopsisluteoalba* (holotype CLZhao 30380): basidiospores (**A**); basidia and basidioles (**B**); dendrohyphidia (**C**); gloeocystidia (**D**); cystidia (**E**); a section of generative hyphae (**F**). Scale bars: 10 µm (**A–F**); 10 × 100.

##### Spores.

Basidiospores ellipsoid, colorless, thin-walled, smooth, IKI–, CB–, (6.5–)7–8 × (4–)4.5–5.5 µm, L = 7.29 µm, W = 4.97µm, Q = 1.47 (n = 30/1).

#### ﻿ Eichleriella biluoxueshanensis

Taxon classificationFungiAuricularialesAuriculariaceae

L. Wang & C.L. Zhao sp. nov.

BF299467-08A4-55BE-B9A4-D984347DF251

856960

Figs 12 , 13 , 14

##### Typification.

China. Yunnan Province • Diqing, Weixi County, Weideng, Songpo, GPS coordinates: 27°05′N, 99°13′E, altitude: 1400 m asl., on the fallen angiosperm branch, leg. C.L., 13 November 2023, CLZhao 34516 (SWFC!).

##### Etymology.

*Biluoxueshanensis* (Lat.) refers to the locality (Biluoxueshan) of the holotype specimen.

##### Basidiomata.

Annual, resupinate, closely adnate, coriaceous, without odor or taste when fresh, up to 8 cm long, 2 cm wide, and 700 μm thick. Hymenophore grandinioid, cream (4A2/3) to buff (4A4) when fresh, buff (4A4) to cinnamon-buff (4/5B4) upon drying. Sterile margin narrow, white (60) to cream (4A2/3), up to 1 mm.

**Figure 12.**
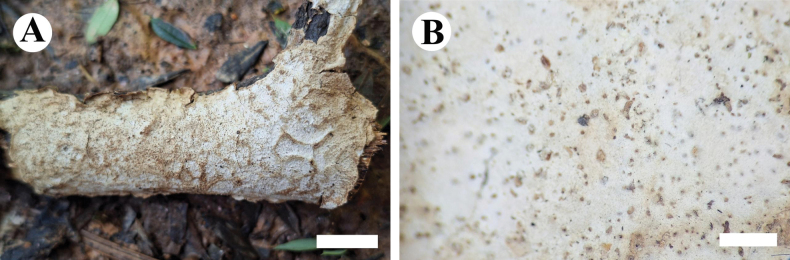
Basidiomata of *Eichleriellabiluoxueshanensis* (holotype CLZhao 34516). Scale bars: 1 cm (**A**); 1 mm (**B**).

##### Hyphal system.

Dimitic; generative hyphae simple-septate, colorless, thin-walled, smooth, rarely branched, interwoven, 3–4 µm in diameter; skeletal hyphae distinctly thick-walled, smooth, unbranched, interwoven, IKI–, CB–, 3–4 µm in diameter; tissues unchanged in KOH.

**Figure 13.**
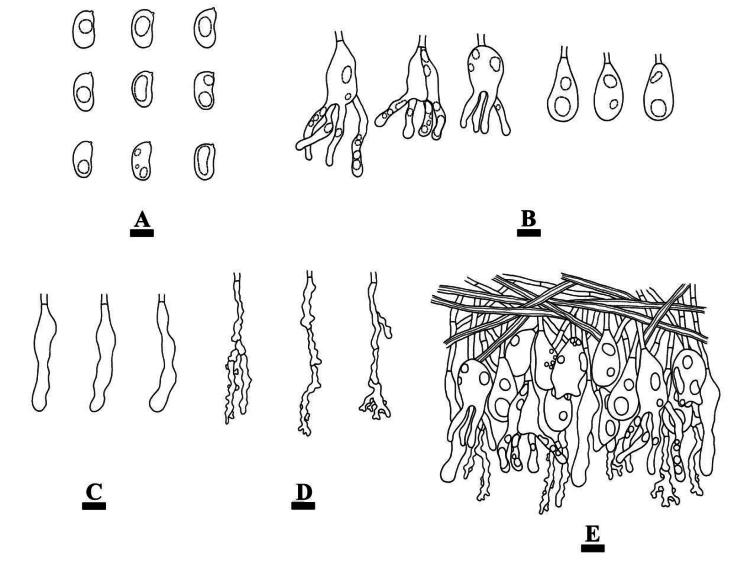
Microscopic structures of *Eichleriellabiluoxueshanensis* (holotype CLZhao 34516): basidiospores (**A**); basidia and basidioles (**B**); cystidia (**C**); hyphidia (**D**); a section of the hymenium (**E**). Scale bars: 10 µm (**A–E**).

##### Hymenium.

Cystidia subclavate, flexuous, colorless, thin-walled, smooth, 40–45.5 × 6–9.5 µm. Basidia narrowly ovoid to ellipsoid, longitudinally septate, four-celled, 21–29 × 11.5–15 µm; basidioles numerous, in shape similar to basidia but smaller. Hyphidia arising from generative hyphae, nodulose, branched, colorless, thin-walled, 58–72.5 × 2.5–4 µm in diameter.

**Figure 14.**
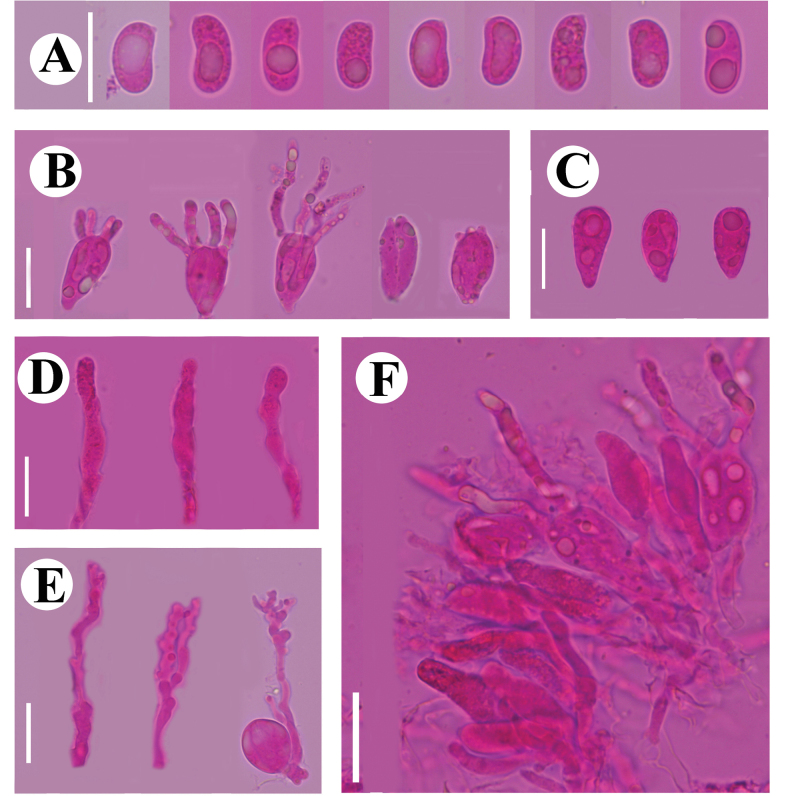
Sections of hymenium of *Eichleriellabiluoxueshanensis* (holotype CLZhao 34516): basidiospores (**A**); basidia (**B**); basidioles (**C**); cystidia (**D**); hyphidia (**E**); a section of the hymenium (**F**). Scale bars: 10 µm (**A–F**); 10 × 100.

##### Spores.

Basidiospores allantoid, colorless, thin-walled, smooth, usually with one or more oil drops, IKI–, CB–, (12–)13.5–17.5(–18) × (6.5–)7–9(–9.5) µm, L = 15.57 µm, W = 7.99 µm, Q = 1.95–2.06 (n = 60/2).

##### Additional specimen examined (paratype).

China. Yunnan Province • Diqing, Weixi County, Zhonglu, Lagaluo, GPS coordinates: 27°9′N, 99°8′E, altitude: 1710 m asl., on the fallen angiosperm branch, leg. C.L. Zhao, 10 October 2023, CLZhao 34793 (SWFC!).

#### ﻿ Gloeohypochnicium yunnanense

Taxon classificationFungiRussulalesGloeohypochnicium

L. Wang & C.L. Zhao sp. nov.

18083A14-BC5B-5C0A-AE73-0C1449626429

856961

Figs 15 , 16 , 17

##### Typification.

China. Yunnan Province • Dehong, Yingjiang County, Tongbiguan Provincial Nature Reserve, GPS coordinates: 25°50′N, 97°36′E, altitude: 1000 m asl., on the dead bamboo, leg. C.L. Zhao, 18 July 2023, CLZhao 30049 (SWFC!).

##### Etymology.

*Yunnanense* (Lat.) refers to the locality “Yunnan Province” of the holotype specimen.

##### Basidiomata.

Annual, resupinate, closely adnate, coriaceous, without odor or taste when fresh, up to 5 cm long, 3 cm wide, and 600 μm thick. Hymenophore smooth, slightly buff (4A4) when fresh, buff (4A4) to cream (4A2/3) upon drying. Sterile margin narrow, white (60) to buff (4A4), up to 1 mm.

**Figure 15.**
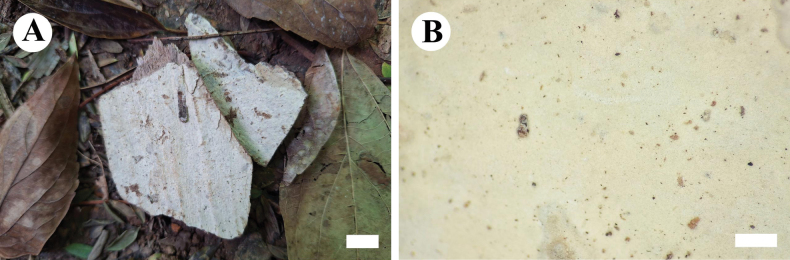
Basidiomata of *Gloeohypochniciumyunnanense* (holotype CLZhao 30049). Scale bars: 1 cm (**A**); 1 mm (**B**).

##### Hyphal system.

Monomitic; generative hyphae with clamp connections, colorless, thick-walled, smooth, branched, interwoven, 1.5–4 µm in diameter; IKI–, CB–, tissues unchanged in KOH.

**Figure 16.**
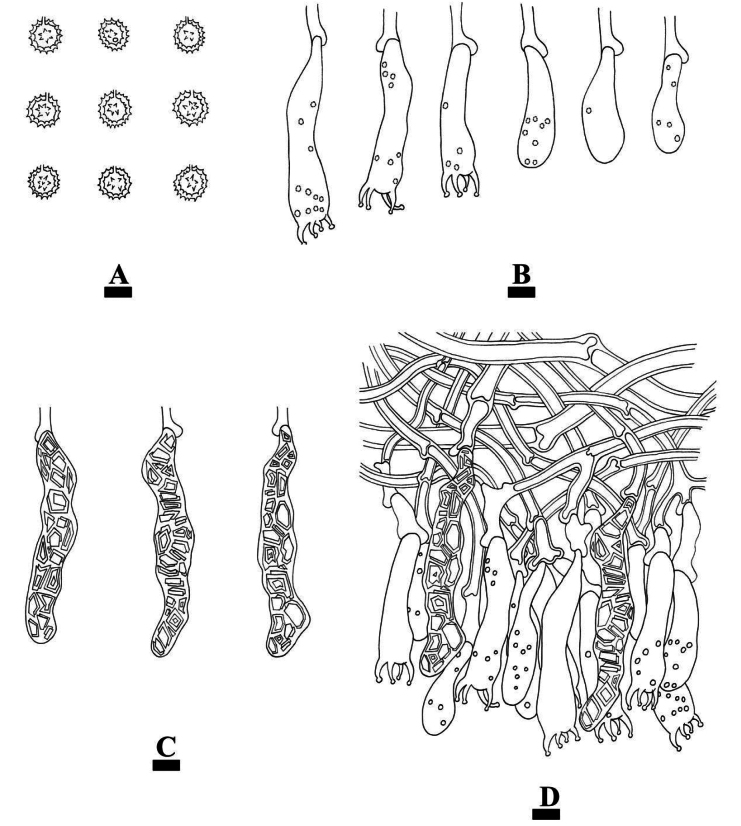
Microscopic structures of *Gloeohypochniciumyunnanense* (holotype CLZhao 30049): basidiospores (**A**); basidia and basidioles (**B**); cystidia (**C**); a section of the hymenium (**D**). Scale bars: 10 µm (**A–D**).

##### Hymenium.

Cystidia subcylindrical to subulate, flexuous, thin-walled, encrusted with whitish to yellowish crystals, 75–115.5 × 11.5–15 µm. Basidia subcylindrical to clavate, slightly flexuous, a basal clamp connection and four sterigmata, 55.5–70 × 9.5–11 µm; basidioles numerous, in shape similar to basidia but smaller.

**Figure 17.**
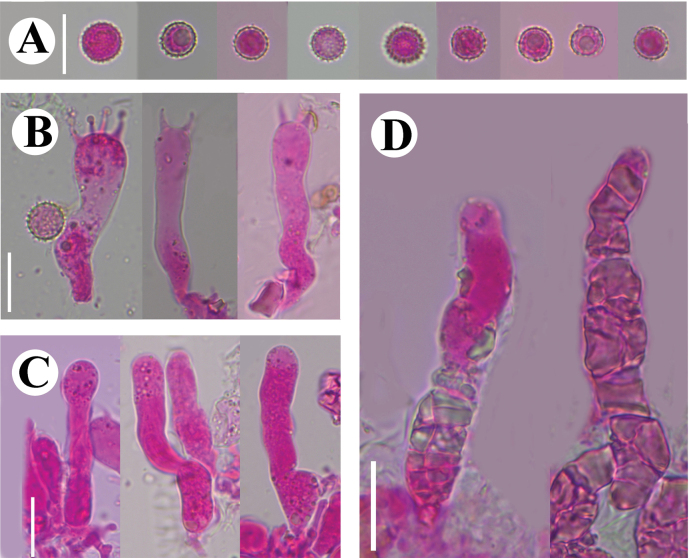
Sections of hymenium of *Gloeohypochniciumyunnanense* (holotype CLZhao 30049): basidiospores (**A**); basidia (**B**); basidioles (**C**); gloeocystidia (**D**). Scale bars: 20 µm (**A–D**); 10 × 100.

##### Spores.

Basidiospores globose, colorless, thick-walled, warted, IKI–, CB–, (9.5–)10–12 × (9.5–)10–11.5 µm, L = 10.91 µm, W = 10.45 µm, Q = 1.04–1.05 (n = 60/2).

##### Additional specimen examined (paratype).

China. Yunnan Province • Dehong, Yingjiang County, Tongbiguan Provincial Nature Reserve, GPS coordinates: 27°52′N, 97°38′E, altitude: 1000 m asl., on dead bamboo, leg. C.L. Zhao, 18 July 2023, CLZhao 30018 (SWFC!).

#### ﻿ Punctularia nigrodontea

Taxon classificationFungiCorticialesPunctulariaceae

L. Wang & C.L. Zhao sp. nov.

374F9664-0CFA-54A3-9106-FB44261E2DDA

856962

Figs 18 , 19 , 20

##### Typification.

China. Yunnan Province • Dehong, Yingjiang County, Tongbiguan Provincial Nature Reserve, GPS coordinates: 25°50′N, 97°36′E, altitude: 1000 m asl., on the angiosperm trunk, leg. C.L. Zhao, 20 July 2023, CLZhao 30592 (SWFC!).

##### Etymology.

*Nigrodontea* (Lat.) refers to the new species having black basidiomata.

##### Basidiomata.

Annual, resupinate to effused-reflexed, adnate but easily separable, gelatinous, without odor or taste when fresh, up to 7 cm long, 3 cm wide, and 600 μm thick. Pileal surface smooth, rigid, fuscous (5/6F5) when fresh, fuscous (5/6F5) to black (51) upon drying; pileal back cushion-shaped grandinioid, rigid, black (51) when fresh, black (51) upon drying. Sterile margin narrow, black (51), up to 1 mm.

**Figure 18.**
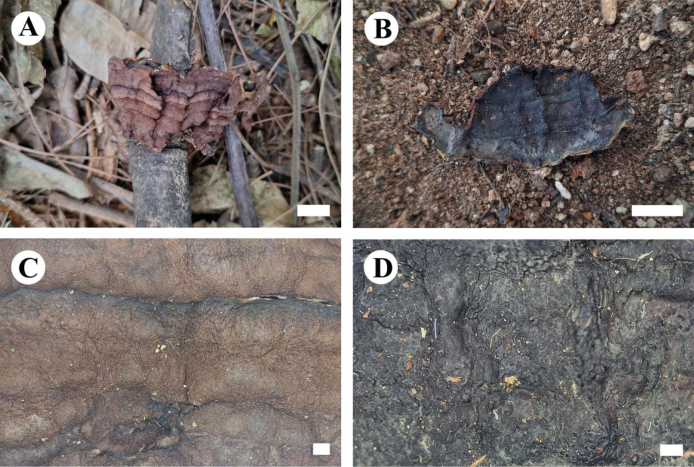
Basidiomata of *Punctularianigrodontea* (holotype CLZhao 30592). Scale bars: 1 cm (**A, B**); 1 mm (**C, D**).

##### Hyphal system.

Monomitic; generative hyphae clamp connections, colorless, thin to thick-walled, smooth, rarely branched, interwoven, 3–7 µm in diameter; IKI–, CB–, tissues unchanged in KOH.

**Figure 19.**
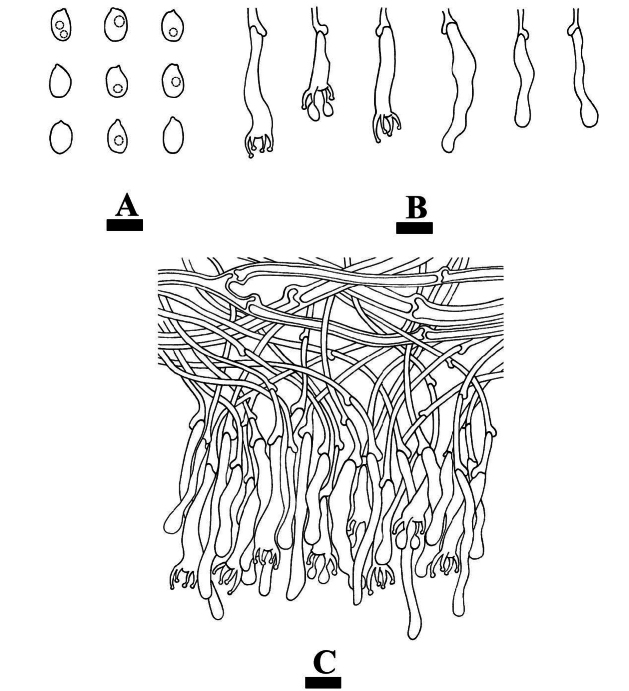
Microscopic structures of *Punctularianigrodontea* (holotype CLZhao 30592): basidiospores (**A**); basidia and basidioles (**B**); a section of the hymenium (**C**). Scale bars: 10 µm (**A–C**).

##### Hymenium.

Cystidia absent. Basidia clavate, flexuous, with a basal clamp connection and four sterigmata, 16–25 × 3–4.5 µm; basidioles numerous, in shape similar to basidia but smaller.

**Figure 20.**
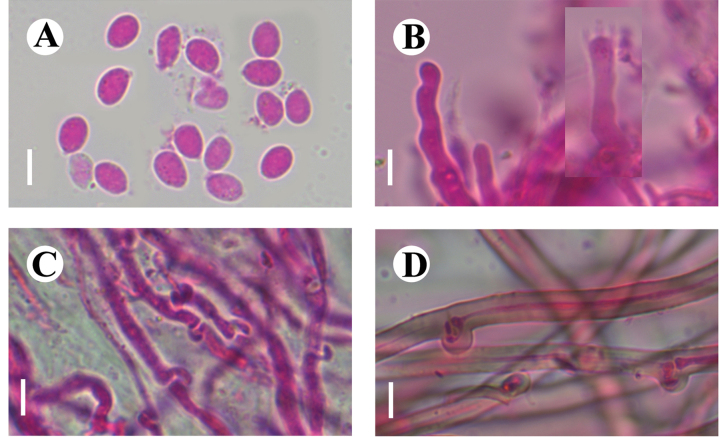
Sections of hymenium of *Punctularianigrodontea* (holotype CLZhao 30592): basidiospores (**A**); basidia and basidioles (**B**); a section of thin-walled generative hyphae (**C**); a section of thick-walled generative hyphae (**D**). Scale bars: 10 µm (**A–D**); 10 × 100.

##### Spores.

Basidiospores ellipsoid, colorless, thin-walled, smooth, IKI–, CB–, 8.5–10(–10.5) × (4.5–)5–6(–6.5) µm, L = 9.24 µm, W = 5.47 µm, Q = 1.69 (n = 30/1).

## ﻿Discussion

For fungal groups that are difficult to identify based on their morphological features, it is believed (and in most cases also proved) that the application of DNA sequences is able to delimit/recognize species much more easily and unequivocally (Wu et al. 2020; Bhunjun et al. 2024; He et al. 2022, 2024; Hyde et al. 2024a, b; Zhao et al. 2024; Zhou et al. 2024). Over time, understanding different aspects of fungi (i.e., taxonomy, diversity, species number) has improved rapidly by incorporating molecular and bioinformatics tools with traditional approaches (Cui et al. 2019; Wang et al. 2021; Hyde et al. 2023; Zhou et al. 2023; Dong et al. 2024a; Wang et al. 2024a; Wijayawardene et al. 2024).

Phylogenetically, the phylogenetic tree (Figs 1, 2) inferred from ITS+nLSU sequences revealed that the new species *Conferticiumalbocremeum* was nested into the family Stereaceae within the order Russulales, in which it was retrieved as a sister to *C.heimii*. However, *C.heimii* is distinguished from *C.albocremeum* by having a grayish orange hymenial surface and smaller basidiospores (5.3–6.2 × 3.5–4.2 µm; Wu 1996). Morphologically, *C.fissuratum* and *C.ravum* are similar to *C.albocremeum* by having finely verrucose, ellipsoid, and thin-walled basidiospores (Bernicchia and Gorjón 2010; Shen et al. 2024). However, *C.fissuratum* differs in its tuberculate, rough, white to cream, and cracking hymenial surface and longer subcylindrical cystidia (37–54.5 × 4–8 µm; Shen et al. 2024), and *C.ravum* differs in its smooth, brownish orange to grayish orange hymenial surface and bigger gloeocystidia (30–60 × 6–15 µm; Bernicchia and Gorjón 2010).

Phylogenetically, the multiple genes with ITS+nLSU analysis (Fig. 3) showed that the new species *Dendrocorticiopsisluteoalba* was nested into the family Punctulariaceae within the order Corticiales, and it is closely related with *D.orientalis*. Morphologically, *D.luteoalba* is similar to *D.orientalis* by having clavate apically with resinous cystidia and clavate to subclavate basidia (Wei et al. 2022). However, *D.orientalis* is delimited from *D.luteoalba* by its finely cracked, grayish ivory hymenial surface and smaller, ellipsoid to ovoid basidiospores (5–7 × 3.2–5.2 µm; Wei et al. 2022).

The phylogenetic tree (Figs 4, 5) inferred from ITS+nLSU sequences revealed that *Eichleriellabiluoxueshanensis* grouped into the genus *Eichleriella* and clustered into the family Auriculariaceae, in which it was grouped with the clade comprising *E.sinensis*. However, *E.sinensis* is distinguished from *E.biluoxueshanensis* by having narrower basidiospores (10.5–16 × 5.5–7 µm; Li et al. 2023). Morphologically, *E.xinpingensis* C.L. Zhao and *E.yunnanensis* Y.L. Deng & C.L. Zhao are similar to *E.biluoxueshanensis* by all having subcylindrical to allantoid, thin-walled, and smooth basidiospores (Liu et al. 2019; Deng et al. 2024a). However, *E.xinpingensis* differs in its soft, leathery to ceraceous, flesh-pink to clay-pink, and covered by blunt-pointed spines hymenial surface and shorter basidia (15–28 × 5–9 µm; Liu et al. 2019), and *E.yunnanensis* differs in its cream to flesh-pink hymenial surface and smaller basidiospores (7.5–11.5 × 3.5–5 µm; Deng et al. 2024a).

The phylogenetic tree (Fig. 1) inferred from ITS+nLSU sequences revealed that *Gloeohypochniciumyunnanense* grouped into the genus *Gloeohypochnicium* and clustered into the order Russulales, in which it was closely related to *G.analogum*. Morphologically, *G.yunnanense* is similar to *G.analogum* in that it has subglobose, thick-walled, and warted basidiospores (Bernicchia and Gorjón 2010). However, *G.analogum* is delimited from *G.yunnanense* by its coriaceous, cream to ochraceous hymenial surface with a fibrillose margin and smaller gloeocystidia (40–60 × 6–10 µm; Bernicchia and Gorjón 2010).

Based on the ITS+nLSU sequence data (Fig. 3), *Punctularianigrodontea* was nested into the family Punctulariaceae within the order Corticiales and grouped with the clade comprising *P.atropurpurascens*, *P.bambusicola*, and *P.strigosozonata*. Morphologically, *P.nigrodontea* is similar to *P.atropurpurascens*, *P.bambusicola*, and *P.strigosozonata* by having smooth, thin-walled, and ellipsoid basidiospores (Bernicchia and Gorjón 2010; Guan et al. 2021). However, *P.atropurpurascens* is delimited from *P.nigrodontea* by having the effuse-reflexed and reddish-brown to dark purplish-brown or bluish hymenial surface and larger basidia (40–65 × 5–6 µm; Guan et al. 2021). *Punctulariabambusicola* differs in its resupinate, tuberculate with rose tints, pink to purple hymenial surface, and smaller basidiospores (6.5–8.5 × 3.5–5 µm; Guan et al. 2021). *Punctulariastrigosozonata* differs in its resupinate to effuse-reflexed basidiomata with a brown, velutinous margin and longer basidia (40–60 × 4–5 µm; Bernicchia and Gorjón 2010).

Fungi are an ancient, diverse, and heterogeneous group of organisms; they can be found in a wide range of habitats, and play key roles in ecosystems as decomposers, mutualists, and pathogens (Dai et al. 2015, 2021; Cui et al. 2023; Wei 2021; Bhunjun et al. 2022). The Yunnan Province is rich in woody plant species, providing excellent substrates for wood-inhabiting fungi (Dong et al. 2023, 2024a; Wang and Cai 2023; Deng et al. 2024b; Wang et al. 2024b; Zhu et al. 2024). Our study is helpful to further understand the species diversity of wood-inhabiting fungal groups in Yunnan and explore their evolutionary relationships.

## Supplementary Material

XML Treatment for Conferticium albocremeum

XML Treatment for Dendrocorticiopsis luteoalba

XML Treatment for Eichleriella biluoxueshanensis

XML Treatment for Gloeohypochnicium yunnanense

XML Treatment for Punctularia nigrodontea

## ﻿Additional information

### Author ORCIDs

Lu Wang https://orcid.org/0009-0004-6274-5953

Changlin Zhao https://orcid.org/0000-0002-8668-1075

### Data availability

All of the data that support the findings of this study are available in the main text.
